# Highly Porous Free-Standing rGO/SnO_2_ Pseudocapacitive Cathodes for High-Rate and Long-Cycling Al-Ion Batteries

**DOI:** 10.3390/nano10102024

**Published:** 2020-10-14

**Authors:** Timotheus Jahnke, Leila Raafat, Daniel Hotz, Andrea Knöller, Achim Max Diem, Joachim Bill, Zaklina Burghard

**Affiliations:** 1 Max Planck Institute for Medical Research, 61920 Heidelberg, Germany; Timotheus.jahnke@mr.mpg.de (T.J.); hotz@mr.mpg.de (D.H.); 2 Institute for Materials Science, University of Stuttgart, 70569 Stuttgart, Germany; leila.raafat@imw.uni-stuttgart.de (L.R.); andrea.knoeller@hahn.schickard.de (A.K.); diem@imw.uni-stuttgart.de (A.M.D.); bill@imw.uni-stuttgart.de (J.B.); 3 Institute for Micro Assembly Technology of the Hahn-Schickard, 70569 Stuttgart, Germany

**Keywords:** aluminum ion batteries, reduced graphene oxide, tin dioxide, 3D electrode materials, mechanical properties

## Abstract

Establishing energy storage systems beyond conventional lithium ion batteries requires the development of novel types of electrode materials. Such materials should be capable of accommodating ion species other than Li^+^, and ideally, these ion species should be of multivalent nature, such as Al^3+^. Along this line, we introduce a highly porous aerogel cathode composed of reduced graphene oxide, which is loaded with nanostructured SnO_2_. This binder-free hybrid not only exhibits an outstanding mechanical performance, but also unites the pseudocapacity of the reduced graphene oxide and the electrochemical storage capacity of the SnO_2_ nanoplatelets. Moreover, the combination of both materials gives rise to additional intercalation sites at their interface, further contributing to the total capacity of up to 16 mAh cm^−3^ at a charging rate of 2 C. The high porosity (99.9%) of the hybrid and the synergy of its components yield a cathode material for high-rate (up to 20 C) aluminum ion batteries, which exhibit an excellent cycling stability over 10,000 tested cycles. The electrode design proposed here has a great potential to meet future energy and power density demands for advanced energy storage devices.

## 1. Introduction

Electrochemical energy storage systems based on lithium ions are nowadays a well-established concept, which enables the application of a broad spectrum of technologies, ranging from microelectronics over portable electronic devices to even electrical cars. Such lithium ion batteries (LIBs) offer excellent energy density and long-term stability. However, the limited lithium resources paired with its high cost and safety hazards make LIBs only a medium-term solution. In the long term, sustainable alternatives, e.g., other metal ion batteries [1], are more favorable. Researchers have been investigating monovalent ion batteries, like sodium and potassium-based electrochemical systems as alternatives [2,3], due to the high abundance and cost efficiency of these elements. However, larger monovalent ions usually have a sluggish diffusivity and lower intercalation voltages at similar energy densities (one electron per ion transfer). To improve the energy density of an active material, electrochemical systems based on multivalent ion exchange have been in discussion among researchers [4,5]. Among them, aluminum-based electrochemical systems have become one of the most promising candidates, owing to the aluminum’s natural abundance, low cost and inherent safety [6,7,8,9]. Moreover, the three-valent aluminum could significantly boost the energy storage capacity compared to the single-valent lithium [10].

Establishing aluminum ion batteries (AIBs) requires the development of suitable electrolytes and tailored electrode materials. Imidazole-based ionic liquids have already been identified as suitable electrolytes, as they exhibit a low inner resistance, high solubility of the aluminum salt AlCl_3_ and good electrochemical stability [11]. The search for cathode materials, however, is still ongoing, and is among the most discussed current topics in this field [7,12]. One fundamental requirement for such a material is its capability to intercalate Al^3+^ ions or chloroaluminate ions at a relatively high operating potential [7]. In this respect, a promising candidate is graphite, with its layered structure that facilitates the access of intercalating ions and provides sufficient space for them. Specifically, it was demonstrated that graphite-based materials immersed in an imidazole-based electrolyte deliver storage capacities of up to 60 mAh g**^−^**^1^ at a high current density of up to 2 A g**^−^**^1^ [13]. Other carbon-based materials, such as carbon nanotubes or graphene/reduced graphene oxide (rGO), have likewise been tested as electrode material for AIBs [7,8,14,15,16,17]. A storage capacity between 60 to 150 mAh g**^−^**^1^ could be achieved due to the reversible intercalation of chloroaluminate ions. Regarding rGO electrodes, electrochemical characterization further revealed an extremely high charging capability of up to 20 A g**^−^**^1^, delivering capacities stable over thousands of cycles [16]. This enhanced electrochemical performance could be ascribed to the material’s pseudocapacitive behavior [18], which arises from the adsorption of chloroaluminate ions onto its surface. The micro/mesoporosity and large specific surface area thereby promote the pseudocapacity, thus enabling a full charging process in minutes or even seconds.

A substantial advantage of pseudocapacitive materials, such as rGO, is that the main contribution to their energy storage capability is attributed to the ion adsorption rather than the bulk ion-diffusion, i.e., de-/intercalation. However, in comparison to LIBs, the intercalation or adsorption of negatively charged aluminum complexes in graphitic materials is still relatively low concerning their gravimetric capacity (<150 mAh g**^−^**^1^) and the energy density (<0.3 Wh g**^−^**^1^) [16]. In addition to graphitic materials, electrochemically active materials, such as sulphur [19,20], sulphides [21,22,23,24,25], and oxides [26,27,28], were investigated for their applicability in AIBs. Even though they showed great potential in terms of their electrochemical storage capacity, they were often lacking in terms of cycling stability or electrical conductivity, leading to capacity fading and short cycle life [12]. Among the oxides, tin oxide (SnO_2_) stands out due to its high specific capacity (434 mAh g**^−^**^1^) [28], good electrical conductivity (1–100 S cm**^−^**^1^) [29,30], and wide availability, although it has only been sparsely investigated. Nevertheless, the combination of carbon-based materials and SnO_2_ has been reported and investigated for LIBs [31,32]. The integration of SnO_2_ nanostructures into a flexible carbon-based matrix has an essential impact of the electrodes performance and can be achieved for example by thermal post treatment [33].

To unite the main properties of pseudocapacitors with those of batteries, thus presenting fast charging rates and good cycling stability with a high storage capacity, the fabrication of hybrid electrodes presents great potential. Moreover, tailoring the hybrid electrode’s microstructure to maximize the specific surface area and shorten the diffusion paths could further boost the synergy of ion adsorption and intercalation. In this work, we therefore united pseudocapacitive rGO nanosheets with electrochemically active SnO_2_ nanoplatelets in highly porous, binder-free aerogel electrodes. Their applicability as cathodes in novel AIBs was tested with respect to their mechanical and electrochemical performance. This work shows that tailoring the porosity and surface area of carbon-based electrode materials in combination with an electrochemically active material, enhances the mechanical stability and the pseudocapacitive performance in AIB.

## 2. Materials and Methods

### 2.1. Fabrication of the Single Components

Graphene oxide was synthesized employing a modified Hummers method [34] Here, 0.5 g graphite flakes (NGS Naturgraphit GmbH) and 0.6 g KNO_3_ (Merck KGaA, Darmstadt, Germany) were added to 23 mL of H_2_SO_4_ (Merck KGaA, Darmstadt, Germany, 98%) in a three-neck, round bottom flask, which is placed in an ice bath. Constant stirring for 10 min was applied to ensure a good dispersion of the graphite flakes in the H_2_SO_4_, whereas afterwards, 3 g KMnO_4_ (Merck KGaA, Darmstadt, Germany) was added, and the temperature adjusted to 35 °C and held for 6 h, while continuing the stirring. Subsequently, 40 mL of dd-H_2_O was added dropwise, and the temperature was increased to 80 °C and held for 30 min. The reaction was interrupted by slowly adding 100 mL dd-H_2_O and 3 mL H_2_O_2_ to the dispersion.

To remove the residual reagents and increase the highly acidic pH (<1), the GO dispersion was centrifuged (SORVALL RC6, Thermo Fischer Scientific, Schwerte, Germany) for 10 min at 17,000 relative centrifugal force. The transparent portion in the flask was decanted after centrifugation, dd-H_2_O added, shortly stirred up and then centrifuged again. This process was repeated at least 10 times, until the pH was above four. The GO sheets, with a sheet size less than 1µm were obtained from IoLiTec, Ionic Liquid Technologies, Karlsruhe, Germany with an initial concentration of 5 mg mL**^−^**^1^.

The SnO_2_ nanoparticles were synthesized using a common hydrothermal approach with SnCl_2_ salt and ammonia as initial reagents [35] 1.36 g SnCl_2_ * 2H_2_O were dissolved in 10 mL dd-H_2_O and 10 mL Ethanol (Merck KGaA, Darmstadt, Germany, p.a.) for 10 min. Subsequently, 24 mL of 0.55 M ammonia solution was added slowly mixture, which turned opaque yellow upon the addition of ammonia. Consequently, the mixture was transferred to a 25 mL Teflon-lined autoclave and hydrothermally treated for 6 h at 120 °C. The material was finally washed alternatingly three times with ethanol and three times with dd-H_2_O to obtain a clean powder.

### 2.2. Ice-Templating and Annealing

To obtain the hybrid composed of rGO and SnO_2_, an aqueous dispersion of the respective GO sheets (66.6 wt%) was mixed with SnO_2_ nanoparticles (33.3 wt%) and sonicated for 10 min in an ultrasonic bath with a 90 W power supply. The dispersion was then transferred into a PTFE mold, which was placed beforehand on the cold finger, and frozen by applying a unidirectional temperature gradient of 18.5 K mm**^−^**^1^ in z-direction. To this end, self-supporting aerogels, with a radius of 4 mm and a height of 8 mm and a porosity of >99.9% were obtained. These self-supporting aerogels were transferred into the freeze drier (L10E, Dieter Piatkowski, Petershausen, Germany), which was cooled down to –50 °C before evacuation. After the removal of the ice crystals, the aerogels were reduced in a quartz tube furnace in Argon (99.95 Ar), ramping with 4 K min**^−^**^1^ to 500 °C, at which they were kept for two hours. The obtained hybrid aerogels had a 41 wt% rGO to 59 wt% SnO_2_ weight ratio and a weight around 1.55 mg ± 0.03 mg, whereas the pure rGO aerogels had a weight of 0.748 mg ± 0.002 mg, obtained from weight measurements on three samples each.

### 2.3. Microstructural Characterization

For microstructural investigations, scanning electron microscopy (Zeiss Ultra 5, Carl Zeiss AG, Oberkochen, Germany) was used. X-ray diffraction was performed on a Bruker D8 system (Bruker Cooperation, MA, USA) using copper K_α_ radiation in the range of 10–90° with 0.01° as step size. The crystallite size was calculated using the Scherrer equation with a form factor of 1 and a Full-Width-Half-Maximum of 1.87 theta around a Bragg Angle of 26.59 deg. Brunauer-Emmett-Teller (BET) tests were performed on a physisorption analyzer (Quantachrome Instruments Autosorb iQ3, Quantachrome/Anton Paar GmbH, Graz, Austria).

### 2.4. Mechanical Characterization

The mechanical measurements were conducted on a Bose Electroforce (TA Instruments, New Castle, DE, USA), specifically the 3220-TA Series III Model, equipped with a 5 mN load cell. To investigate the porous foams, glass plates were glued on the bottom and the cross head and the sample was placed between them. The measurement was performed first with a compression step with a strain rate of 0.015 mm s**^−^**^1^ up to 75% compression, followed by a release step with a strain rate of 0.015 mm s**^−^**^1^ down to 0% compression.

### 2.5. Electrochemical Characterization

The cell assembly was performed in an argon-filled glovebox (Labmaster SP, MBraun, Garching, Germany) and VWR connections made of PTFE with an inner diameter of 10.5 mm were used as cell housing. To avoid corrosion on the contacts, molybdenum rods were milled from pure molybdenum (99.95 at.% Mo) rods to until they had a diameter of 10.5 mm and fit tightly into the housing. Additionally, the contacts were wrapped with PTFE sealing tape, to exclude any influence of oxygen. The respective aerogel was placed on one side of the contact and six glass fiber separators (Grade 934-AH, Whatman, Merck KGaA, Darmstadt, Germany) with a diameter of 11 mm on top. 1-Ethyl-3-methylimidazolium chloride mixed with aluminum chloride in the ratio of 1:1.3 (IoLiTec, Ionic Liquid Technologies, Karlsruhe, Germany) was determined to be a suitable electrolyte. The separators and the aerogel were soaked with electrolyte (0.2 mL) and compressed using the other Mo contact as stamp. As counter electrode aluminum foil (99.99 at.% Al) was used. Galvanostatic charge/discharge tests were performed at a current density of 100, 1000 and 2000 mA g**^−^**^1^ in the voltage range of 0.2–2.0 V for the composite and 0.2 to 2.2 V for the rGO aerogel. Prior to testing, the cells sat for at least two hours to completely saturate the aerogel with electrolyte. Cyclic voltammetry was performed in a voltage window of 0.02–2.2 V, with a sweep rate of 0.1, 1, 10, 20, 50 and 100 mV s**^−^**^1^ for both aerogels. All measurements were performed on electrochemical test stations (VMP300, Biologic, Seyssinet-Pariset, France).

## 3. Results and Discussion

### 3.1. Fabrication of the Porous, Binder-Free rGO/SnO_2_ Aerogels

Graphene oxide (GO) sheets were fabricated using a modified Hummers’ method [34], resulting in sheet sizes ranging from 2 to 20 µm (Supporting Figure S1a). The SnO_2_ nanoplatelets were synthesized by a hydrothermal approach [35]. Their dimensions were determined by scanning electron microscopy (SEM), revealing a thickness of a few nanometers and a lateral dimension of several tens of nanometers, with a tendency to form agglomerates with a size of 100 nm (Supporting Figure S1b). 

**Figure 1:**
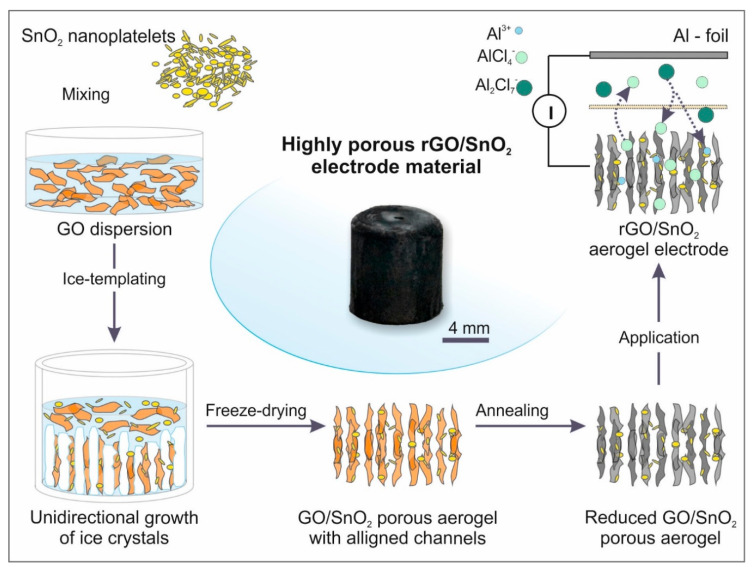
Schematic illustration of the fabrication process of free-standing highly porous rGO/SnO_2_ aerogels and their application as a cathode material in AIBs.

The hybrid electrode was fabricated by adding SnO_2_ nanoplatelets to an aqueous dispersion of the GO sheets. Soft sonication treatment was thereby performed to ensure a homogenous distribution (Figure 1) and exfoliation of GO sheets. Subsequently, the components were co-assembled by an ice-templating process. Ice crystals propagate thereby in a unidirectional manner along the applied temperature gradient in the z-direction, and thus restrict the solid load between them (Supporting Figure S2). Their subsequent sublimation, i.e., freeze-drying, leaves a highly porous, channel-like microstructure behind, in which the columnar pores are the replica of the removed ice crystals [36]. Such an ice-templating technique enables aligned, continuous channels throughout the whole height of the cylindrical aerogel. For the performance evaluation, aerogels composed solely of GO were fabricated, using the same concentration of GO in aqueous dispersion as that used for the hybrid electrode aerogel. 

**Figure 2:**
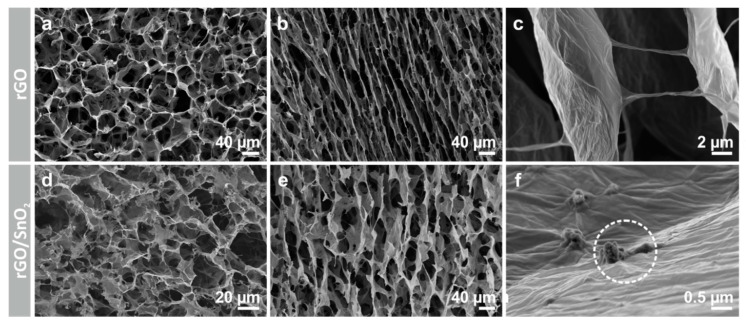
SEM images of the microstructures of rGO and rGO/SnO_2_ aerogels (**a**,**d**) top view and (**b**,**e**) side view of the channels. Detailed view of the (**c**) GO walls and (**f**) the SnO_2_ nanoplatelets embedded in the walls.

The final step towards the fabrication of rGO/SnO_2_ hybrid electrodes for AIBs (Figure 1) is annealing the aerogel to thermally reduce the GO. In this step, it is crucial to choose a slow heating rate to avoid fast gas evolution, which would lead to structural damage. To verify the structural preservation after annealing of the aerogels, pristine and hybrid, the microstructure was investigated by SEM in its original (Supporting Figure S3) and reduced state (Figure 2). To that end, a honeycomb-like pore structure is observed at the cross-section of the as-prepared and reduced aerogels (Figure 2a,d and Supporting Figure S3a,d), with a pore width in the range of 20 to 40 µm. From the longitudinal cross sections, the aligned pore channels are notable and a wall porosity in the range of tens of micrometers is revealed (Figure 2b,e and Supporting Figure S3b–e). The latter arises from local inhomogeneities of solid load between the ice crystals [37], coupled with the lateral size of the GO sheets, which is up to one magnitude smaller than the diameter of the ice crystals/channels. Moreover, a connection between the channels is observed in the longitudinal section (Figure 2c). The microstructural features identified for the aerogels are typical for ice-templated carbon materials [38,39,40]. Additionally, regarding the hybrid, agglomerates of SnO_2_ nanoplatelets could be detected, which are wrapped between the GO sheets, asserting the co-assembly of both materials (Figure 2f and Supporting Figures S3f and S4). The striking similarity of the as-prepared and reduced aerogels’ microstructures therefore ensured the minimal impact of the thermal treatment.

**Figure 3:**
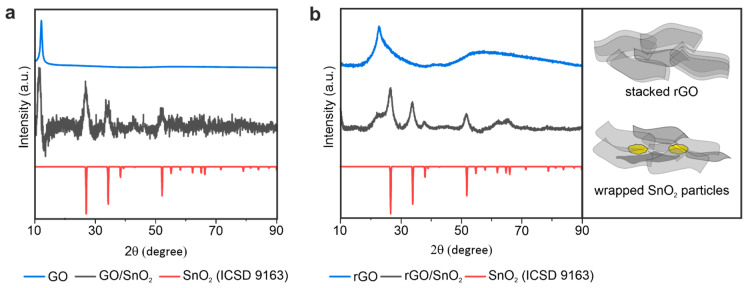
XRD pattern of the (**a**) GO/SnO_2_ and (**b**) rGO/SnO_2_ hybrid aerogel with a schematic presentation of the rGO sheets arrangement with and without SnO_2_ particles.

However, upon thermal treatment, a weight loss of 26% occurred, which is assigned to the removal of oxygen-containing functional groups and their reaction to gaseous CO and CO_2_ [41]. A hybrid composition of 41 wt% rGO and 59 wt% SnO_2_ is thereby obtained as deducted from thermogravimetric analysis (Supporting Figure S5). Moreover, highly conductive rGO is achieved due to the restoration of the sp^2^-hybridization characteristic of graphene [42]. The removal of the oxygen-containing functional groups is further accompanied by a decrease in interlayer distance of neighboring sheets [43]. The powder X-ray diffraction (XRD) patterns of the hybrid aerogels, as-prepared and reduced, revealed a shift of the reflection at 11° for GO to 24° for rGO (Figure 3a,b). The reflection at around 11° originates from stacked GO, as it correlates with the pristine GO material. The shift therefore corresponds to a decrease in interlayer distance from 0.8 nm to 0.4 nm, which is close to the value of the interlayer distance in graphite (0.33 nm) [44]. This correlates to the results obtained by Raman, where an increase in the D- to G-band ratio is observed (Supporting Figure S6). Additionally, a peak broadening is observed for rGO, which correlates to a small crystallize size in the sheets [16]. Similarly, the same shift upon reduction is observed for the hybrid as a shoulder at 24° next to the reflection of SnO_2_, indicating a decrease in layer distance of overlapping rGO sheets, which are located in the walls of the aerogel. However, the intensity of the shoulder is significantly lower and broader, compared to SnO_2_, indicating a smaller diffracted crystal volume of rGO stacks as schematically presented in Figure 3b. This reduction in intensity is correlated with the decreased number of stacked rGO sheets, because SnO_2_ particles are embedded between them hindering the restacking of rGO single sheets during removal of oxygen containing surface groups (see schematic in Figure 3b). XRD analysis shows that the SnO_2_ nanoplatelets are nanocrystalline and exhibit a rutile structure, known as a good intercalation host [36,45,46,47]. After reduction, these rutile reflections of SnO_2_ become more pronounced, indicating a crystal growth of the originally agglomerated nanostructured platelets into larger crystals (Supporting Figure S4). The crystallite size of the SnO_2_ nanostructures was calculated using the Scherrer equation as 6.33 nm.

Brunauer–Emmett–Teller (BET) measurements were performed on the pristine as well as the hybrid aerogels to determine their surface area and mesoporosity, as these features are known to highly influence the electrochemical performance of the active material [16]. A large surface area of 221.3 m^2^ g**^−^**^1^, in the case of the hybrid rGO/SnO_2_, was thereby determined, 60% of that of the pristine rGO aerogel (367.9 m^2^ g**^−^**^1^). The presence of mesoporosity was also verified by the obtained N_2_ adsorption–desorption isotherms, exhibiting a hysteresis form (Supporting Figure S7) [48]. This result is in good agreement with the presented microstructure (Figure 2b,e), revealing the location of the mesopores within the cell walls. However, the mesoporosity of the pristine aerogels is almost 2-fold that of the hybrid aerogels, as concluded from the cumulative pore volume of 0.47 cm^3^ g**^−^**^1^ in the case of the pristine and 0.24 cm^3^ g**^−^**^1^ in the case of the hybrid aerogel (Supporting Figure S7b). However, considering the significantly higher density of the hybrid aerogel (5.0 g cm**^−^**^3^) an even larger cumulative pore volume fraction is achieved. Furthermore, the overall porosity *P* = 1 − *ρ*_s_/*ρ* *100% is estimated to 99.9% for both aerogels, pristine and hybrid, based on the density of carbon *ρ* = 2.26 g cm**^−^**^3^ and that of the aerogel *ρ*_s_ [44]. The scaffold structure, with its channel-like pores, is the major contributor to this high porosity. Notably, the tailored microstructure of the rGO/SnO_2_ aerogels, combining the channel-like network of pores (macroporosity) with the cell wall mesoporosity, is promising. Specifically, the latter facilitates ion diffusion and enables the access to an increased number of intercalation and adsorption sites, while a homogenous distribution of the electrolyte throughout the electrode along the applied potential gradient is assured by the macroporosity [23].

### 3.2. Mechanical Performance of the rGO/SnO_2_ Aerogels

**Figure 4:**
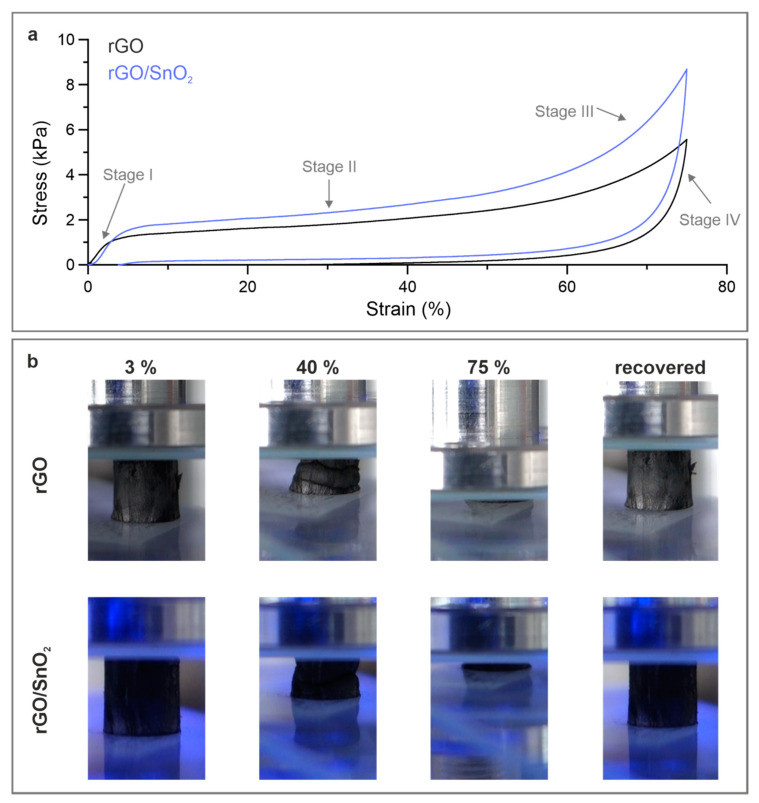
Mechanical performance of the rGO and rGO/SnO_2_ aerogels under compression. (**a**) The compressive stress–strain curve with (**b**) the corresponding in situ images of the different compression states.

The aerogels need to be compressed when implementing them in the battery cell as a free-standing cathode material. Mechanical testing of the aerogels was thereby conducted by means of compression, to ensure their structural integrity upon cell assembly and electrochemical analysis. The density and microstructure of the aerogels, specifically the degree of ordering and the alignment of the rGO sheets, thereby play a crucial role, defining the mechanical response. To this end, the size of the rGO sheets contributes immensely to the elastic mechanical performance. Sheets larger than 20 µm provide structural recovery upon deformation and a strength one order of magnitude higher than those smaller than 2 µm [38]. However, the size of the sheets also influences the electrochemical performance, whereas for smaller edge-rich sheets, an increased capacity in AIBs is obtained due to the additional intercalation sites at the edges [49]. Therefore, an optimal sheet size with sufficient mechanical stability and optimal electrochemical performance is crucial. The investigation of the rGO and rGO/SnO_2_ aerogels (Supporting Figure S8), with an initial GO sheet size smaller than 2 µm, revealed a poor mechanical performance (Supporting Figure S9). Therefore, rGO and rGO/SnO_2_ aerogels fabricated with a sheet size larger than 20 µm were investigated. Specifically, they would provide high elasticity and mechanical stability, similar to elastomeric materials, and yet sufficient edges for facilitated ion de-/intercalation. In particular, the continuous packing of the rGO within a cell wall and their interconnection promote their strength and superelasticity [38]. The shape of the obtained compressive stress–strain curves of the aerogels, whether pristine or hybrid (Figure 4a), indicate four typical deformation stages of cellular materials [50,51]. Specifically, stage I (strain < 5%) correlates to the elastic deformation, stage II (strain up to 50%) to cell wall buckling and stage III (strain up to 75%) to the densification of the aerogels [52]. Stage IV represents the recovery of the aerogels, referring to the flexibility of the structure with the rGO sheets. The macroscopic deformation of the aerogels is displayed in Figure 4b through digital images representative of the different stages (Supporting Movie S1 and S2). The elastic deformation of the aerogels is characterized by a Young’s modulus similar for both aerogels, 0.41 ± 0.06 kPa for the pristine and 0.42 ± 0.09 kPa for the hybrid aerogel, with a slight increase in the compressive strength (Supporting Table S1). Considering that the presented aerogels exhibit a porosity of ~99.9%, these values are in good agreement to those obtained for other graphene/graphene oxide aerogels [52].

Notably, the rGO/SnO_2_ aerogels show a superior recovery of 95.5% compared to pristine rGO aerogels (53.3%) (Figure 4a and Supporting Movie S3), allowing a reversible compression over a wide strain range with only a slight (3.5%) permanent deformation (Figure 4b). The latter is ascribed to defects in the aerogels’ cell walls, such as micro-cracks [38], while the high recovery is attributed to the flexibility of the rGO sheets as well as the tailored structure-design, allowing significant energy absorption. The superior recovery of the hybrid aerogels is assumed to originate from the wrapping, or anchoring, of the SnO_2_, which interconnects neighboring rGO sheets and interlock upon proceeding mechanical deformation. Such performance renders these hybrid aerogels as an ideal electrode material with a high mechanical stability, which maintains its flexibility and a certain porosity even under very high compression states. The accommodation of larger ions is thereby possible while the structural integrity of the electrodes is preserved.

### 3.3. Electrochemical Performance of the rGO and rGO/SnO_2_ Aerogels

**Figure 5:**
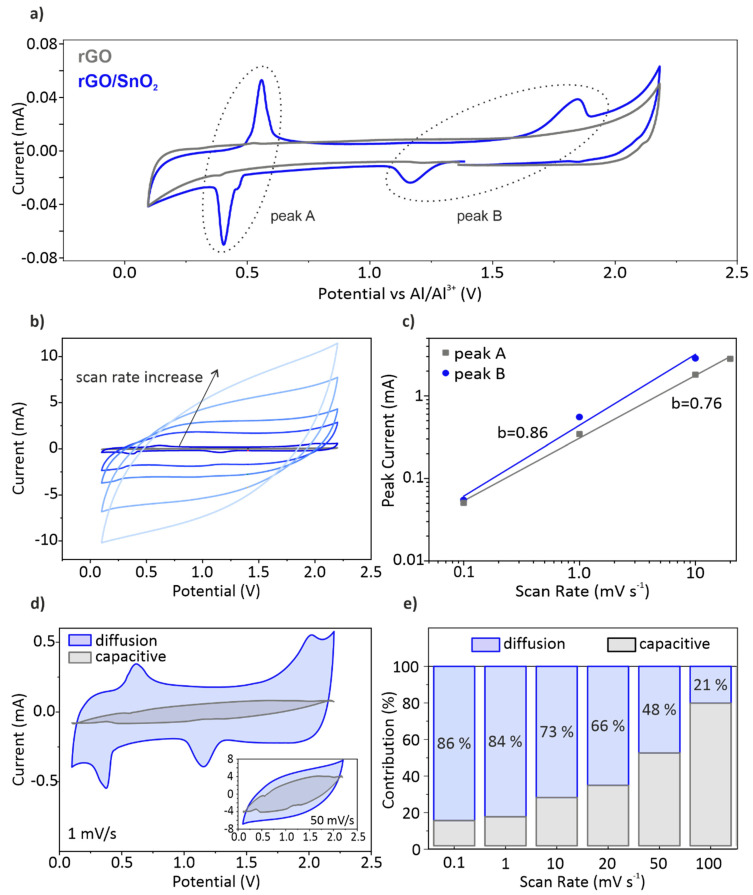
(**a**) Cyclic voltammograms (CV) of the rGO/SnO_2_ hybrid electrode in comparison with the pristine rGO electrode at 0.1 mV s^−1^ (2nd cycle). (**b**) CVs at 0.1, 1, 10, 20, 50 and 100 mV s**^−^**^1^ for the rGO/SnO_2_ hybrid electrode (2nd cycle). (**c**) Plot of the anodic peak current at the different scanning rates for b-value determination of peak A and peak B. (**d**) The contribution of the diffusion and capacitive controlled current response at 1 mV s**^−^**^1^ and 50 mV s**^−^**^1^ (inset). (**e**) The Faradaic and non-Faradaic contribution to the whole capacity (capacitive vs. diffusion-controlled energy storage) of the rGO/SnO_2_ composite for all scanning rates, ranging from 0.1 mV s**^−^**^1^ up to 100 mV s**^−^**^1^.

The ultra-light, free-standing and binder-free rGO and rGO/SnO_2_ aerogels were electrochemically tested by directly soaking them with electrolyte and compressing them into discs upon cell assembly. The impact of additives used in conventional slurry-based electrodes on the electrochemical performance is thereby excluded, allowing to investigate the individual contribution of rGO and SnO_2_ within the hybrid material. The electrodes were evaluated in the voltage window between 0.2 V and 2.2 V vs. Al/Al^3+^. Figure 5a shows the corresponding cyclic voltammetry (CV) curves. For the pristine rGO aerogel, a plateau between 0.15 V and 1.7 V is observed [53]. This current–voltage behavior, where the current is predominantly linearly proportional to the voltage rate, is typical for non-Faradaic energy storage. In contrast, the hybrid rGO/SnO_2_ electrode displays an anodic and a corresponding cathodic peak around 0.5 V (peak A), which is correlated with the de-/intercalation of Al^3+^ in SnO_2_ [28]. Upon intercalation of the Al^3+^ into the rutile crystal structure of SnO_2_, the tetra-valent Sn(IV) is reduced to divalent Sn(II), resulting in Al_x_SnO_2_ [28]. Whereas, the amount of intercalated Al^3+^ ions, denoted as x, reaches a maximum of 0.6. Interestingly, the CV curves of the hybrid electrode exhibits an additional anodic peak at 1.8 V and a corresponding cathodic peak at 1.3 V (peak B), which is not related to the intercalation into SnO_2_. The origin of the peak is hypothesized to be the de-/intercalation of AlCl_4-_ at the interface between the SnO_2_ particles and the rGO sheets.

This hypothesis is supported by results obtained for a freeze-dried rGO/SnS_2_ composite [23]. They observed a peak with a large shift between positive and negative scan direction at a similar intercalation voltage.

Owing to the high surface area of the tailored aerogel electrodes, a large electrochemical double layer is formed. A significant capacitive contribution is thereby achieved analogous to the pristine rGO electrode (Figure 5a). The energy storage process is therefore a combination of de-/intercalation of the ions into SnO_2_ as well as between rGO/SnO_2_ and their adsorption on the rGO surface. Hence, an interplay between Faradaic and non-Faradaic processes occurs during the charging and discharging processes, using the active material to its utmost.

To distinguish the contribution of the diffusion-controlled processes to the overall energy storage from that of the pseudo-capacitive effect, CV measurements with scan rates ranging from 0.1 mV s**^−^**^1^ up to 100 mV s**^−^**^1^ were conducted (Figure 5b). At scan rates, up to 10 mV s**^−^**^1^, the current peaks shift with a noticeable increase in peak separation. Upon increasing the scan rate to 100 mV s**^−^**^1^, however, the peaks are not detectable anymore, and the energy storage process shifts to a mainly non-Faradaic characteristic. By plotting the peak current against the scan rate in a double-logarithmic plot (Figure 5c), indication of the predominant process can be shown [54]. The slope b of the thereby obtained linear regression follows *I* = a *ν*^b^, where *I* corresponds to the peak current, *ν* to the scanning rate and a to the y-intersect. Values for b approaching unity are characteristic for capacitance-controlled processes, while values around 0.5 indicate diffusion-controlled processes [55]. The b-value is thereby determined as 0.76 for peak A and 0.86 for peak B, implying that both processes contribute. However, the higher value for peak B indicates a more capacitance-controlled process, denoting more surface-controlled kinetics. This additional peak arises therefore not only from intercalation of ions between SnO_2_ and rGO, but mostly from their adsorption at the surface. To determine the contribution percentage of each process type, whether diffusion-controlled or surface-controlled, the corresponding current at different potential points is evaluated [18]. The cyclic voltammograms at 1 and 50 mV s**^−^**^1^ (Figure 5d) present the contribution of the different processes. A notable decrease of the capacitive current at the potential corresponding to the de-/intercalation processes, peak A and B, can be seen. Moreover, an increase of the capacitive contribution at the higher scan rate is apparent, comprising more than 50% at 50 mV s**^−^**^1^. The contribution of the diffusion and capacitive processes for the various scanning rates is shown in Figure 5e. That of capacitive ion adsorption onto the surface of the electrodes increases with the charging rates, as anticipated. Surprisingly, the contribution of diffusion-controlled processes is dominant at a scanning rate of up to 50 mV s**^−^**^1^. This behavior unites the main properties of a capacitive-controlled supercapacitor and a diffusion-controlled battery, combining high power and energy densities. Compared to a recently published work about graphite–graphite dual ion batteries using the same electrolyte, the diffusion-controlled contribution for the hybrid aerogel at the same scanning rate of 1 mV s**^−^**^1^ is more than two-fold increased [56]. This significantly increases the results from the synergy of nanosized SnO_2_ particles wrapped by rGO sheets and the tailored microstructure of the hybrid aerogel electrode. Due to the small size of the SnO_2_ particles, de-/intercalation is enabled at very high scanning rates of 100 mV s**^−^**^1^, delivering a sizable contribution to the capacity (Figure 5e). The energy storage process is therefore optimized, simultaneously exploiting the large surface area of 221.3 m^2^ g**^−^**^1^ induced by the tailored microstructure of the rGO-based aerogel electrode and the electrochemically active nanometer sized SnO_2_ particles. The unidirectional channels, which originate from ice-templating, enable fast infiltration of electrolyte even at a compressed state. Additionally, they decrease the electron pathway and increase the high rate performance of the electrode [57]. This enables a supercapacitor-like performance at very high charging rates, coupled with comparatively large capacity values, thus overcoming the limitation in either energy or power density typically found in batteries or supercapacitors, respectively.

**Figure 6:**
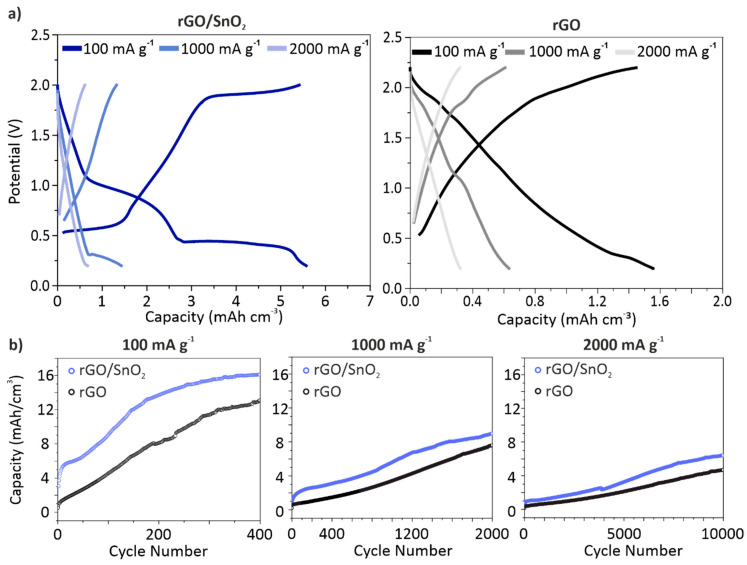
(**a**) Charge–discharge profiles of the hybrid rGO/SnO_2_ (left) and pristine rGO (right) electrodes at the 10th cycle. (**b**) Rate performance of electrodes at a current density of 100, 1000 and 2000 mA g**^−^**^1^. Their volumetric energy storage capacity was calculated for a compressed electrode with a thickness of 100 µm.

To further evaluate the rate performance of the hybrid aerogels in comparison to the pristine material, galvanostatic charge–discharge profiles with a charging rate of 2 C, 20 C and 40 C were conducted (Figure 6). A specific gravimetric capacity of the hybrid aerogel of 50 mAh g**^−^**^1^ was obtained for a current density of 100 mA g**^−^**^1^ (2 C). The impact of the SnO_2_ nanoparticles addition on the electrochemical performance of the aerogels is concluded from the comparison of their volumetric capacities. The significant difference in gravimetric densities of the materials in question is thereby excluded.

From the charge–discharge profiles (Figure 6a) of the hybrid electrode a large plateau around 0.5 V is observed, which correlates to peak A (Figure 5a). By increasing the charging rate, the length of the plateau decreases, equivalent to our findings from the CV analysis. However, the less pronounced plateau, corresponding to peak B (Figure 5a), disappears at higher charging rates. Additionally, a pseudocapacitive behavior is observed for the hybrid electrode, analogous to the pristine electrode. These findings corroborate the results obtained from the cyclic voltammetry analysis, demonstrating the synergy of the energy storage processes present in the hybrid electrodes. The volumetric capacity delivered by the hybrid electrode is accordingly higher by 23% than that of the pristine electrode. From the cell life investigation (Figure 6b), the same trend of the capacity evolution is observed for both electrodes. The increase of the capacities can therefore be attributed to the activation of the rGO and its subsequent reduction [41]. Furthermore, the initially high efficiency of 105% supports this conclusion (Supporting Figure S10). Moreover, by increasing the charging rate lower capacities are attained, reaching a capacity of 16.1 mAh cm**^−^**^3^ at 2 C and only 8.9 and 6.4 mAh cm**^−^**^3^ at 20 and 40 C, respectively. Notably, the hybrid electrodes deliver higher volumetric capacities than the pristine rGO electrodes, over 30% at the various charging rates. Compared to other cathode materials for AIBs the hybrid aerogel electrodes deliver adequate energy densities (20–50 Wh kg**^−^**^1^) at notably high power densities (810–100 W kg**^−^**^1^), as shown in the Ragone plot (Supporting Figure S11). On this basis, the benefit of the SnO_2_ nanoparticle loading on the porous channel-like rGO aerogel is validated. Furthermore, the well-defined nanostructure of SnO_2_ hinders the pulverization of the particles, leading to an excellent cycling stability over 10,000 cycles.

**Figure 7:**
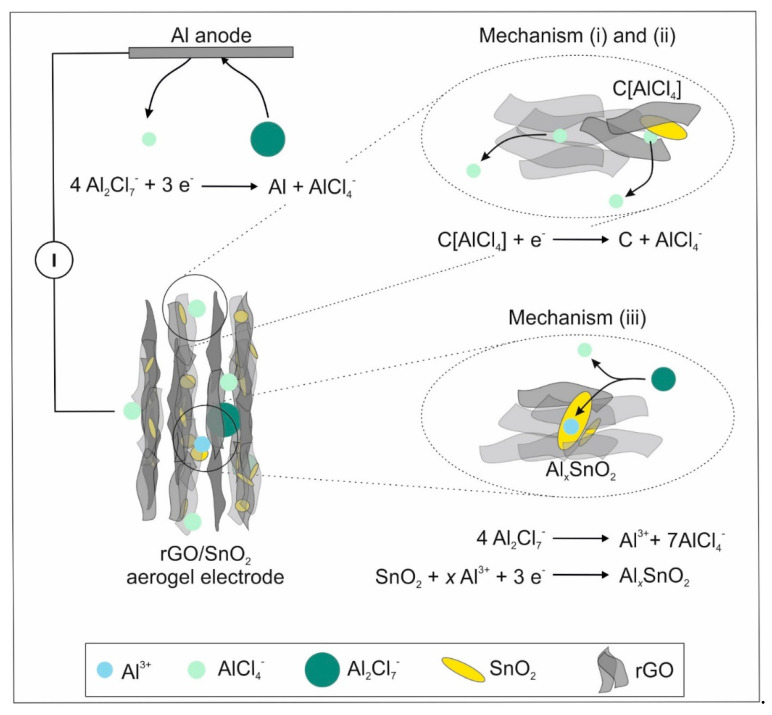
A schematic representation of the rGO/SnO_2_ aerogel electrode in a half-cell configuration (left), where the three different energy storage mechanisms occurring at the cathode are presented with the corresponding simplified chemical reactions (right).

The electrochemical performance of the hybrid electrodes observed here can be attributed to three different energy storage mechanisms (Figure 7).

(i) The non-Faradaic contribution is partially correlated with the electrochemical double layer capacitance observed for rGO sheets with crystallite sizes below 10 nm [16]. The chloroaluminate anions are thereby adsorbed at the surface of rGO sheets.
(ii) Peak B is hypothesized to correlate with the intercalation of AlCl_4-_ between SnO_2_ and rGO. The SnO_2_ nanoparticles embedded in the rGO aerogel, provide sufficient space for intercalation, as they increase the cumulative pore volume fraction. When considering graphitic materials, intercalation involving AlCl_4-_ results in a characteristic peak around 2 V [13,57]. Additionally, the SnO_2_ nanocrystals distort the graphitic structure, which could facilitate the intercalation and thus lowers the voltage. This mechanism contributes further to the pseudocapacitive behavior of the hybrid aerogel electrode.
(iii) The Faradaic contribution entails the de-/intercalation of Al^3+^ into SnO_2_ (peak A), which was similarly observed for SnO_2_/C cathodes [28].

## 4. Conclusions

We fabricated a highly porous, free-standing rGO/SnO_2_ aerogel as a binder-free cathode for AIBs. The tailored microstructure of these aerogels provides a synergy of mechanical stability and enhanced electrochemical performance. It is characterized by aligned channels, the walls of which comprise rGO sheets and embedded SnO_2_ nanoparticles, resulting in high flexibility with a significant structural recovery upon mechanical compression, up to 95.5%. Furthermore, the here-achieved integration of nanosized SnO_2_ particles into the rGO aerogel creates a synergistic effect, where pseudocapacitive and diffusion-controlled energy storage mechanisms simultaneously contribute to the deliverable capacity and prolong the battery life, over 10,000 cycles. An enhancement of the volumetric capacity by 23% is thereby achieved, as opposed to its rGO aerogel counterpart. In addition, a notably high power density of 810 W kg**^−^**^1^ was achieved for the hybrid aerogels, comparable or superior to that of state-of-the art hybrid electrodes for AIBs. A step towards solving the current predicament in mobile energy devices is thereby made, as a combination of high power and high energy density, achievable by a single energy storage device, is attained. Our findings therefore provide new insights into the conceptual design of high-performance hybrid electrodes with pseudocapacitive behavior for AIBs.

## Supplementary Materials

The following are available online at https://www.mdpi.com/2079-4991/10/10/2024/s1, Figure S1: SEM images of (**a**) a GO sheet, prepared by the modified Hummers Method (size between 2 to 20 µm). (**b**) SnO_2_ nanoplatelets, prepared by a hydrothermal approach; Figure S2: Schematic illustration of the (**a**) ice-templating setup and (**b**) the evolution of unidirectional ice crystal growth, Figure S3: SEM images of the microstructures of GO and GO/SnO_2_ aerogels (**a**,**d**) top view and (**b**,**e**) side view of the channels. Detailed view of the (**c**) GO walls and (**f**) the SnO_2_ nanoplatelets embedded in the walls, Figure S4: Highly magnified SEM images of the (**a**) GO/SnO_2_ and (**b**) rGO/SnO_2_ hybrid aerogel. In both cases, the sheets wrap the SnO_2_ nanoplatelets and hold them in place, Figure S5: Thermogravimetric analysis of the annealing process at 500 °C in argon and its corresponding mass loss of 26% and the determination of the rGO to SnO_2_ ratio in synthetic air at 1100 °C, showing that 41 wt% of the composite is comprised of rGO, whereas 59 wt% is comprised of SnO_2_, Figure S6: Raman Spectra of GO/SnO_2_ (grey) and rGO/SnO_2_ (red) samples, where the D- and G-bands are visible, Figure S7: Isotherm obtained by N2 adsorption and desorption curves of rGO and rGO/SnO_2_ aerogels and their respective cumulative pore volume, Figure S8: SEM images of the microstructures of rGO and rGO/SnO_2_ aerogels, which were fabricated using a GO dispersion with much smaller sheet sizes. (**a**,**d**) Top view and (**b**,**e**) side view of the channels. Detailed view of the (**c**) GO walls and (**f**) the SnO_2_ nanoplatelets embedded in the walls, Figure S9: Mechanical performance of the rGO and rGO/SnO_2_ aerogels, which were fabricated using a GO dispersion with smaller sheet sizes (<2 µm), under compression. (**a**) The compressive stress-strain curve with (**b**) the corresponding in-situ images of the different compression states, Figure S10: Efficiency plot of both the pristine and the hybrid aerogel, Figure S11: Ragone plot of the hybrid rGO/SnO_2_ aerogel in comparison to the most recent literature in the field of AIB’s. Supplementary Movie 1. High magnification movie of the compression test on a rGO/SnO_2_ scaffold.Supplementary Movie 2. High magnification movie of the compression test on a rGO scaffold. Supplementary Movie 3. Practical demonstration of the scaffolds flexibility. Supplementary Table S1. Mechanical properties of rGO and rGO/SnO_2_ aerogels. Supplementary Table S2: Mechanical properties of rGO and rGO/SnO_2_ aerogels, which were fabricated using a GO dispersion with much smaller sheet sizes.
