# Supplementary material for: Highly Porous Free-Standing rGO/SnO2 Pseudocapacitive Cathodes for High-Rate and Long-Cycling Al-Ion Batteries
Source: Nanomaterials (Basel). 2020 Oct 14;10(10):2024. doi: 10.3390/nano10102024 (PMC7602191; doi:10.3390/nano10102024)
Supplement: Supplementary file 1 [file nanomaterials-10-02024-s001.pdf]

nanomaterials

Supplementary Materials

Highly Porous Free-Standing rGO/SnO_2 Pseudocapacitive Cathodes for High-Rate and Long-Cycling Al-Ion Batteries

Timotheus Jahnke [1], Leila Raafat [2], Daniel Hotz [1], Andrea Knöller [2,3], Achim Max Diem [2], Joachim Bill [2] and Zaklina Burghard [2,*]

1 Max Planck Institute for Medical Research, 61920 Heidelberg, Germany; Timotheus.jahnke@mr.mpg.de (T.J.); hotz@mr.mpg.de (D.H.)
2 Institute for Materials Science, University of Stuttgart, 70569 Stuttgart, Germany; leila.raafat@imw.uni-stuttgart.de (L.R.); andrea.knoeller@hahn.schickard.de (A.K.); diem@imw.uni-stuttgart.de (A.M.D.); bill@imw.uni-stuttgart.de (J.B.)
3 Institute for Micro Assembly Technology of the Hahn-Schickard, 70569 Stuttgart, Germany
* Correspondence: zaklina.burghard@imw.uni-stuttgart.de; Tel.: 0049 711 685 61958

Received: 14 September 2020; Accepted: 12 October 2020; Published: 14 October 2020

Supplementary Materials: The following are available online at www.mdpi.com/xxx/s1,

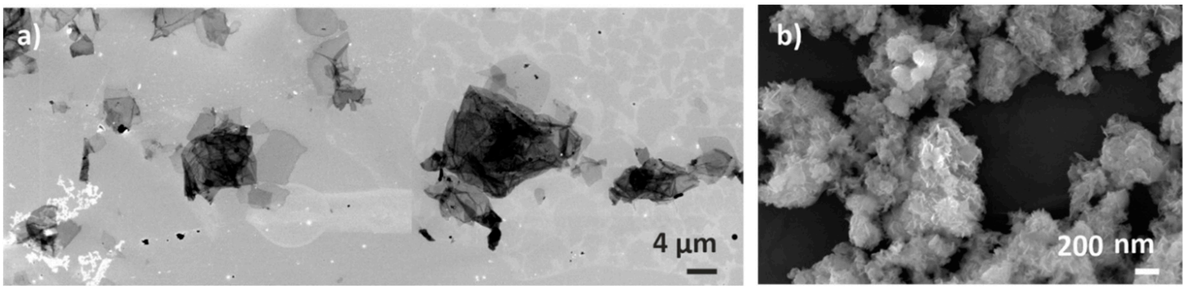

Figure S1. SEM images of (**a**) a GO sheet, prepared by the modified Hummers Method (size between 2 to 20 µm). (**b**) SnO_2 nanoplatelets, prepared by a hydrothermal approach.

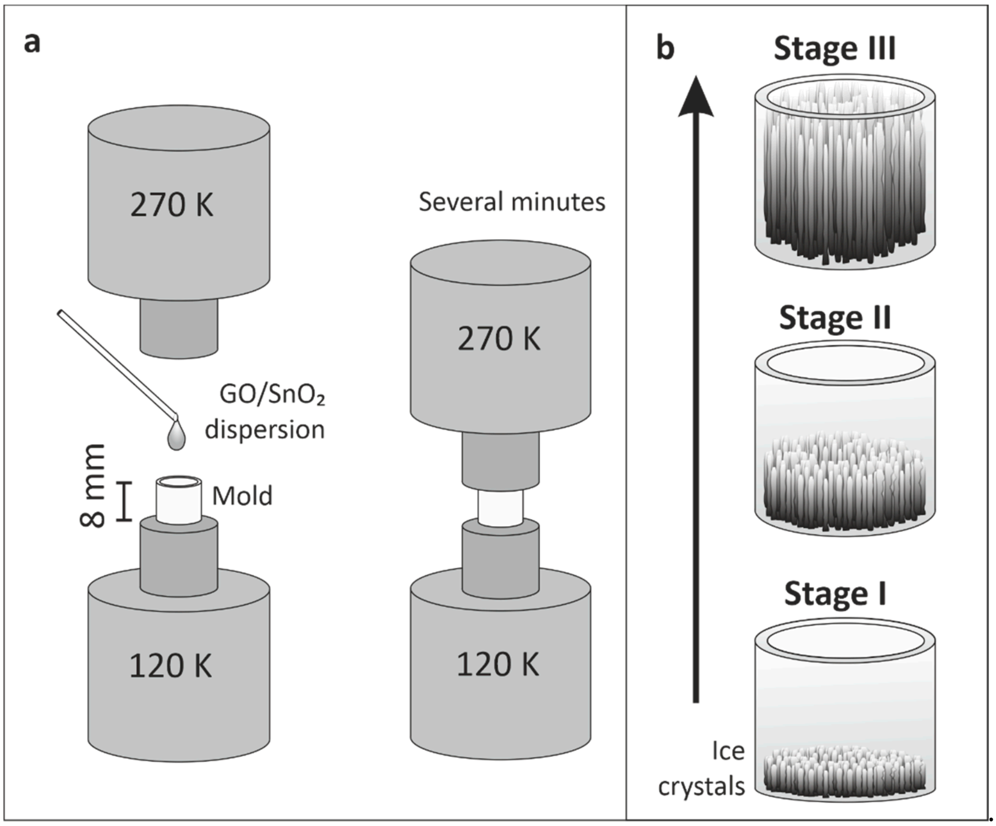

Figure S2. Schematic illustration of the (**a**) ice-templating setup and (**b**) the evolution of unidirectional ice crystal growth.

Ice-Templating

In general, applying this technique enables the fabrication of highly porous, mechanically stable aerogels. When using the self-build setup schematically displayed in Supporting Figure S2a, the ice-templating process can be precisely controlled. The two copper cold fingers at the top and bottom of the mold are cooled with liquid nitrogen. As the cold fingers are equipped with heating rings, the temperature of each cold finger can be individually set, enabling precise control over the temperature gradient in between. A PTFE mold is placed between the cold fingers, making it possible to fabricate cylindrical aerogels with a height and diameter of eight mm. When the desired temperature gradient is set, the dispersion can be filled into the mold and the upper cold finger will be lowered onto the mold.

The ice-templating process itself is schematically depicted in Supporting Figure S2b, where a freezing gradient of 150 K between both cold fingers induces a fast unidirectional crystal growth, as soon as the liquid comes into contact with the cooled cold finger (Stage I). Hereby, the ice crystals nucleate and grow along the gradient, while the solid dispersed material is expelled at the ice crystal interface. Due to the large gradient, the ice front moves from the bottom to the top of the mold, yielding columnar ice crystals as a result. Such columns aligned along the freezing direction mechanically support the aerogel, resulting in an increased Young's modulus, compared with foam frozen using different methods [1]. Upon further solidification (Stage II), the initial temperature gradient decreases, promoting ice crystal growth perpendicular to the temperature gradient. In this stage, the solid load between the ice crystals becomes compacted. Finally, in Stage III, the mold is completely frozen, and ice crystals have grown to the top of the mold.

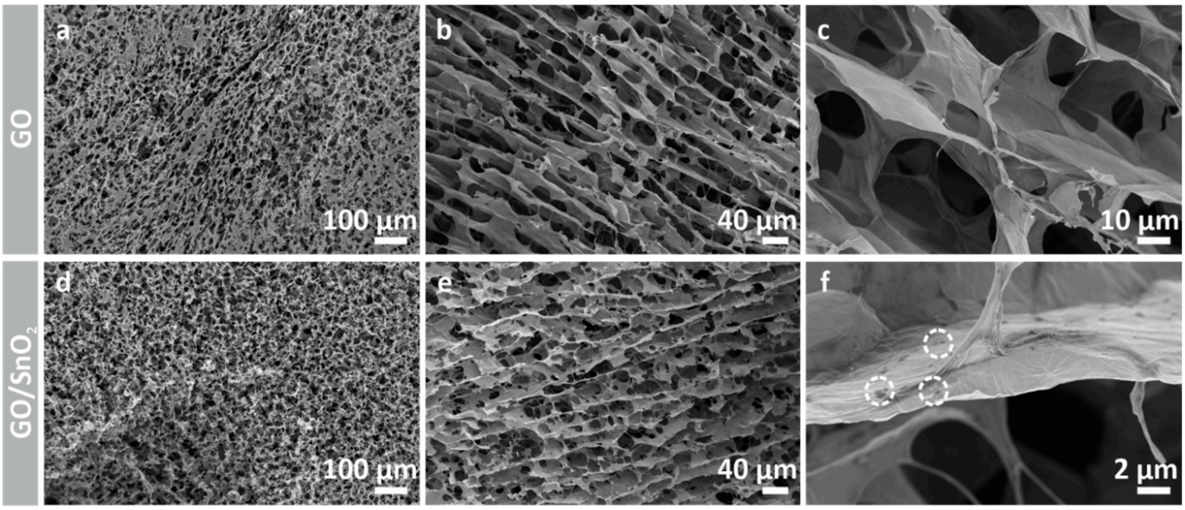

Figure S3. SEM images of the microstructures of GO and GO/SnO_2 aerogels (**a**,**d**) top view and (**b**,**e**) side view of the channels. Detailed view of the (**c**) GO walls and (**f**) the SnO_2 nanoplatelets embedded in the walls.

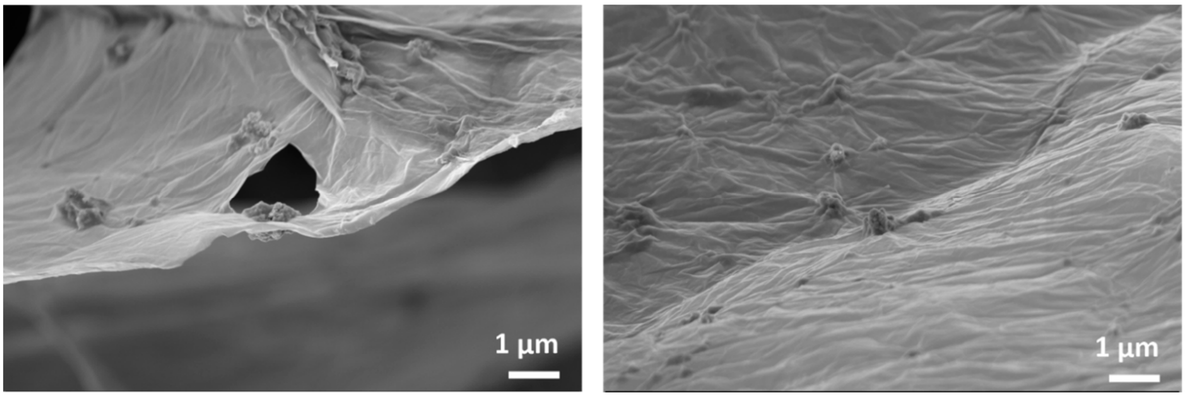

Figure S4. Highly magnified SEM images of the (**a**) GO/SnO_2 and (**b**) rGO/SnO_2 hybrid aerogel. In both cases, the sheets wrap the SnO_2 nanoplatelets and hold them in place.

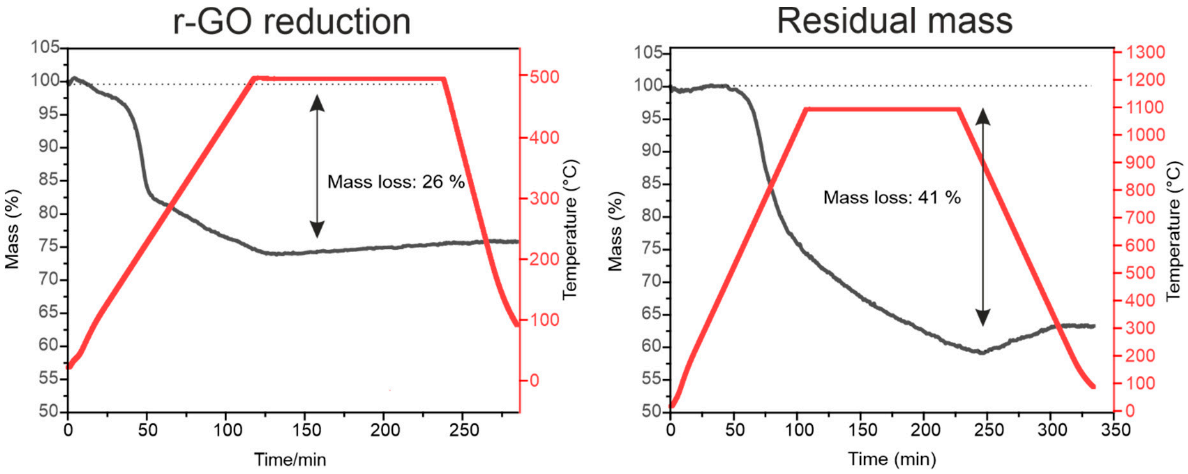

Figure S5. Thermogravimetric analysis of the annealing process at 500 °C in argon and its corresponding mass loss of 26% and the determination of the rGO to SnO_2 ratio in synthetic air at 1100 °C, showing that 41 wt% of the composite is comprised of rGO, whereas 59 wt% is comprised of SnO_2.

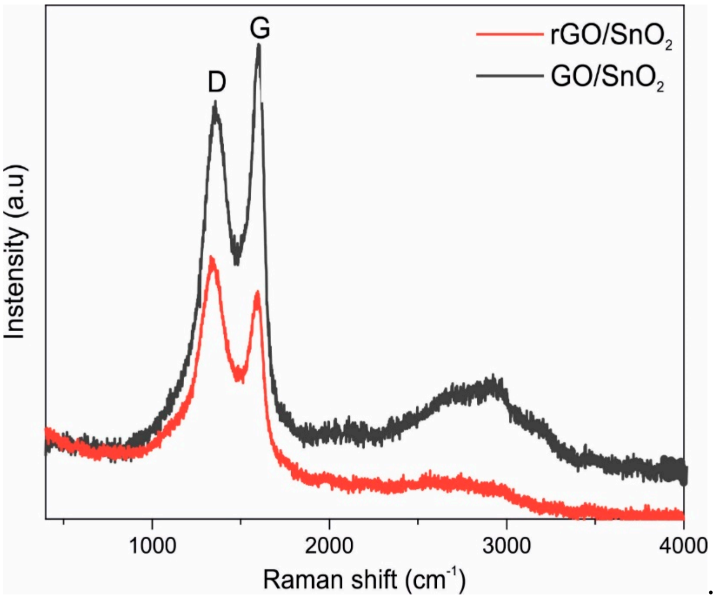

Figure S6. Raman Spectra of GO/SnO2 (grey) and rGO/SnO2 (red) samples, where the D- and G-bands are visible.

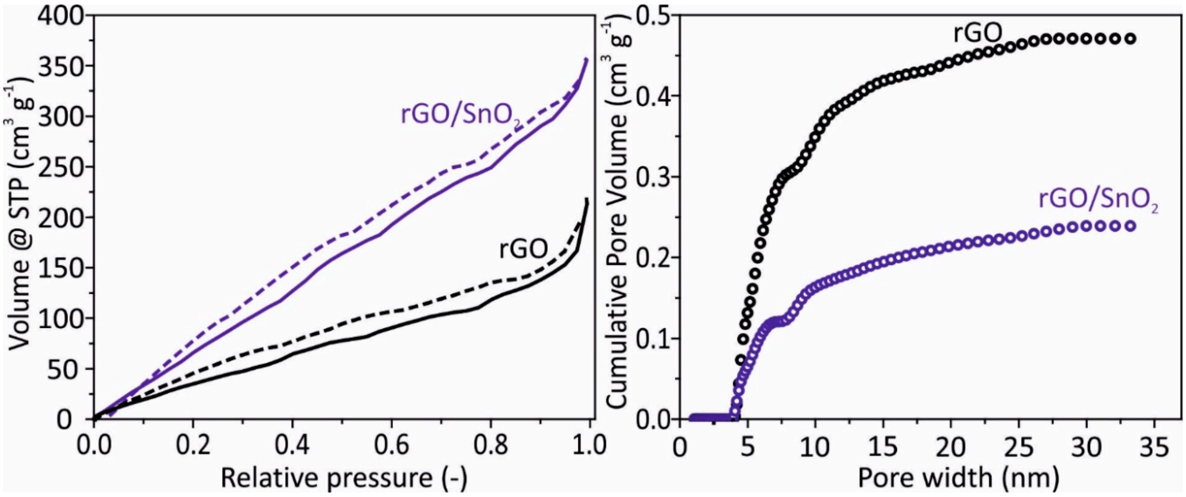

Figure S7. Isotherm obtained by N2 adsorption and desorption curves of rGO and rGO/SnO2 aerogels and their respective cumulative pore volume.

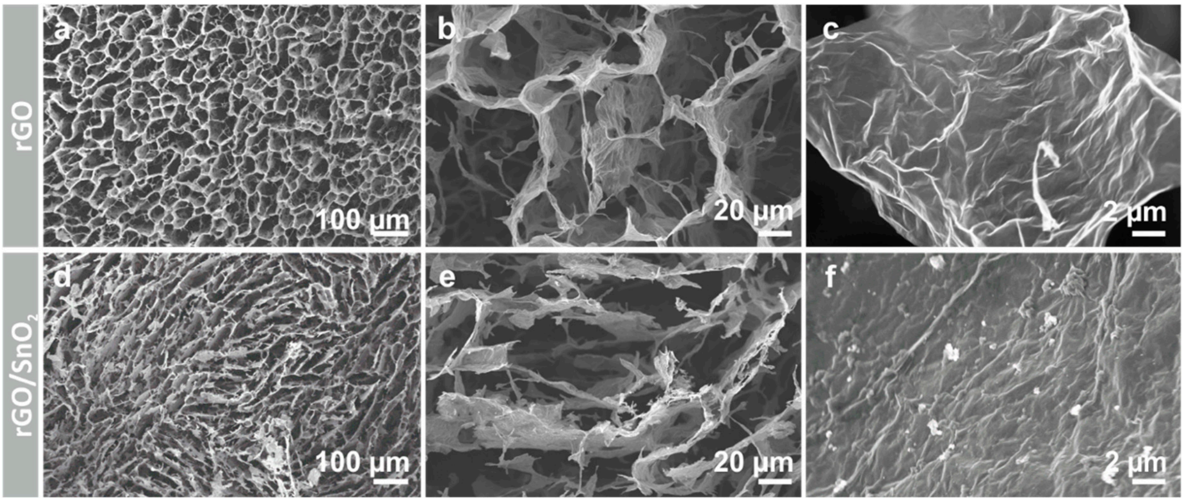

Figure S8. SEM images of the microstructures of rGO and rGO/SnO_2 aerogels, which were fabricated using a GO dispersion with much smaller sheet sizes. (**a,d**) Top view and (**b,e**) side view of the channels. Detailed view of the (**c**) GO walls and (**f**) the SnO_2 nanoplatelets embedded in the walls.

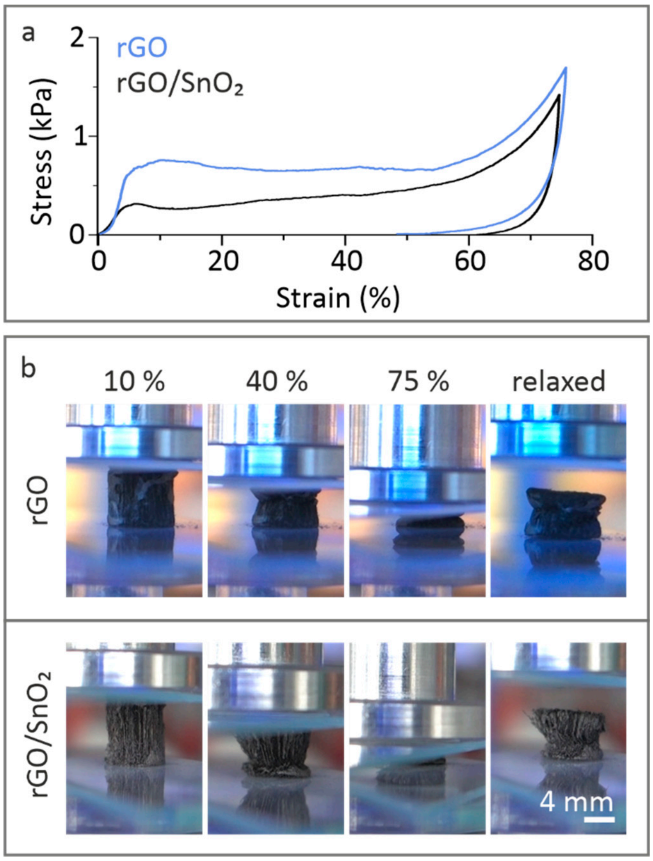

Figure S9. Mechanical performance of the rGO and rGO/SnO_2 aerogels, which were fabricated using a GO dispersion with smaller sheet sizes (< 2 µm), under compression. (**a**) The compressive stress–strain curve with (**b**) the corresponding in situ images of the different compression states.

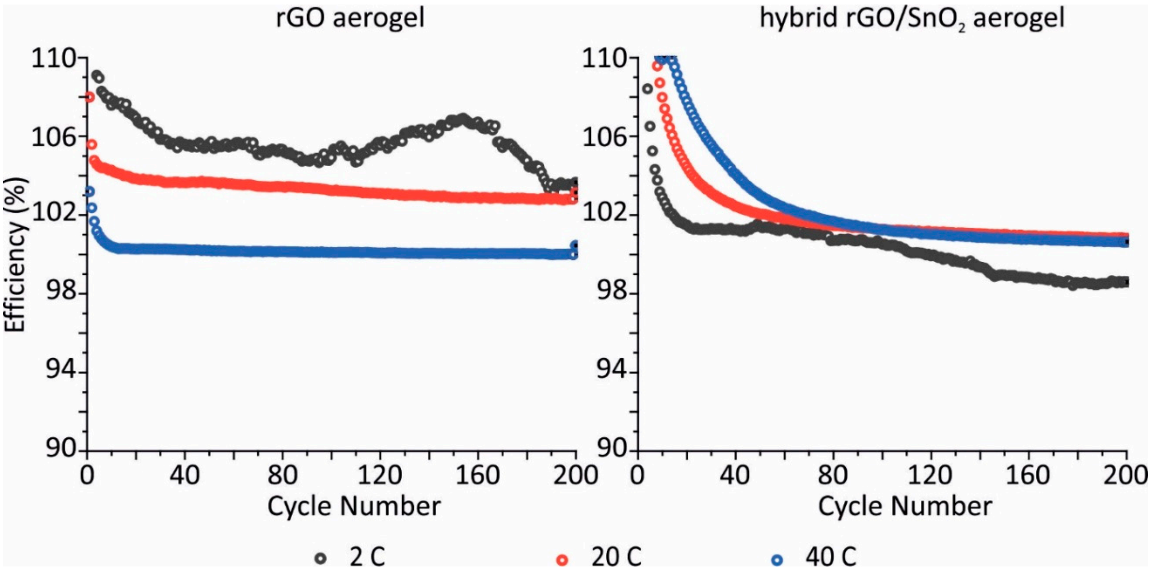

Figure S10. Efficiency plot of both the pristine and the hybrid aerogel.

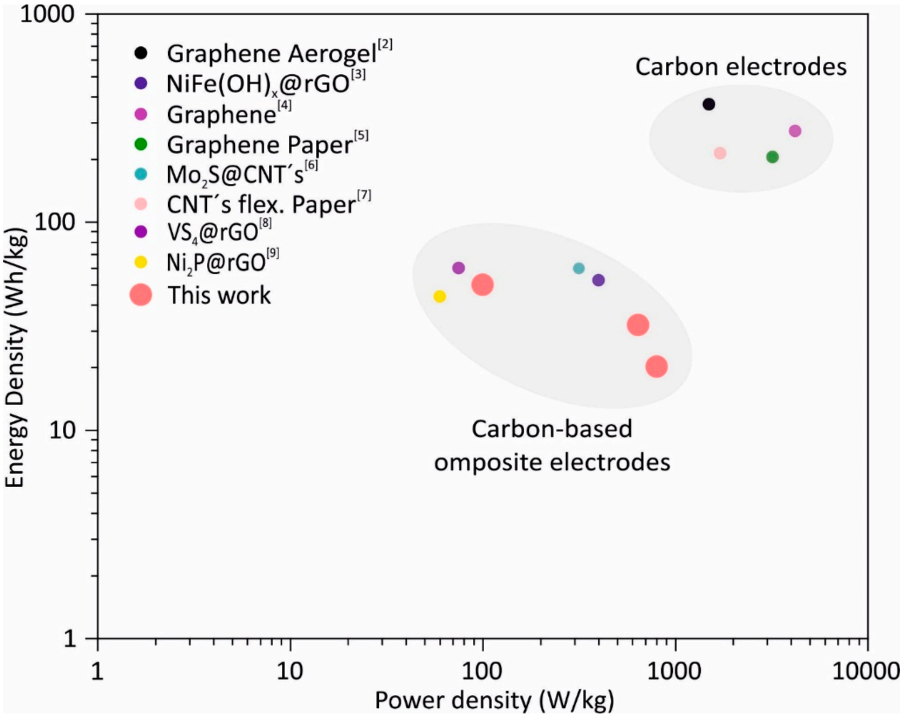

Figure S11. Ragone plot of the hybrid rGO/SnO_2 aerogel in comparison to the most recent literature in the field of AIB's [2–9].

Supporting Table S1: Mechanical properties of rGO and rGO/SnO2 aerogels.

	Young's modulus [kPa]	Compressive strength [kPa]	Recovery [%]
**rGO**	0.41 ± 0.06	1.39 ± 0.19	53.3
**rGO/SnO_2**	0.42 ± 0.09	1.55 ± 0.13	95.5

Supporting Table S2: Mechanical properties of rGO and rGO/SnO2 aerogels, which were fabricated using a GO dispersion with much smaller sheet sizes (see also Supporting Figure S1b).

	Young's modulus [kPa]	Compressive strength [kPa]	Recovery [%]
**rGO**	0.26 ± 0.14	1.06 ± 0.35	36
**rGO/SnO_2**	0.08 ± 0.03	0.31 ± 0.03	20

Supporting Information References:

1. Z. Zhao, M. Sun, W. Chen, Y. Liu, L. Zhang, N. Dongfang, Y. Ruan, J. Zhang, P. Wang, L. Dong, Y. Xia, H. Lu, Sandwich, Vertical-Channeled Thick Electrodes with High Rate and Cycle Performance, *Advanced Functional Materials* **2019**, 29, 1809196.
2. H. Huang, F. Zhou, X. Shi, J. Qin, Z. Zhang, X. Bao, Z.-S. Wu, Graphene aerogel derived compact films for ultrafast and high-capacity aluminum ion batteries, *Energy Storage Materials* **2019**, 23, 664.
3. Y. Du, S. Zhao, C. Xu, W. Zhang, P. Li, H. Jin, Y. Zhang, Z. Wang, J. Zhang, Novel Ni–Fe-Layered Double Hydroxide Microspheres with Reduced Graphene Oxide for Rechargeable Aluminum Batteries, *Energy Technology* **2019**, 7, 1900649.
4. A. Ejigu, L.W. Le Fevre, K. Fujisawa, M. Terrones, A.J. Forsyth, R.A.W. Dryfe, Electrochemically Exfoliated Graphene Electrode for High-Performance Rechargeable Chloroaluminate and Dual-Ion Batteries, *ACS Applied Material Interfaces* **2019**, 11, 23261.
5. Q. Zhang, L. Wang, J. Wang, C. Xing, J. Ge, L. Fan, Z. Liu, X. Lu, M. Wu, X. Yu, H. Zhang, B. Lu, Low-temperature synthesis of edge-rich graphene paper for high-performance aluminum batteries, *Energy Storage Materials* **2018**, 15, 361.
6. W. Yang, H. Lu, Y. Cao, B. Xu, Y. Deng, W. Cai, Flexible Free-Standing MoS2/Carbon Nanofibers Composite Cathode for Rechargeable Aluminum-Ion Batteries, *ACS Sustainable Chemistry & Engineering* **2019**, 7, 4861.
7. Y. Hu, S. Debnath, H. Hu, B. Luo, X. Zhu, S. Wang, M. Hankel, D.J. Searles, L. Wang, Unlocking the potential of commercial carbon nanofibers as free-standing positive electrodes for flexible aluminum ion batteries, *Journal of Materials Chemistry A* **2019**, 7, 15123.
8. X. Zhang, S. Wang, J. Tu, G. Zhang, S. Li, D. Tian, S. Jiao, Flower-like Vanadium Suflide/Reduced Graphene Oxide Composite: An Energy Storage Material for Aluminum-Ion Batteries, *ChemSusChem* **2018**, 11, 709.
9. J. Tu, M. Wang, X. Xiao, H. Lei, S. Jiao, Nickel Phosphide Nanosheets Supported on Reduced Graphene Oxide for Enhanced Aluminum-Ion Batteries, *ACS Sustainable Chemistry & Engineering* **2019**, 7, 6004.
